# Early Marriage, Preterm Birth, and School Dropout: An Intergenerational Cycle of Risk?

**DOI:** 10.1002/ajhb.70177

**Published:** 2025-12-15

**Authors:** Jonathan C. Wells, Qisty Noviyanti, Akanksha A. Marphatia, Emeline Rougeaux

**Affiliations:** ^1^ Childhood Nutrition Research Centre, Population Policy and Practice Department UCL Great Ormond Street Institute of Child Health London UK; ^2^ Department of Disease Control London School of Hygiene and Tropical Medicine London UK

**Keywords:** child marriage, early marriage, education, intergenerational, preterm, schooling

## Abstract

**Background:**

Across generations, girls' early marriage recurs in high‐risk groups; however there is poor understanding of how behavior and biology interact in this context. We hypothesized an intergenerational cycle of risk, linking early marriage, preterm birth, and school dropout, and evaluated evidence for specific components of this cycle in low‐/middle‐income countries.

**Methods:**

We conducted a systematized review, searching articles published from 2000 to 2025. We tested four hypotheses. H1: early marriage is associated with preterm birth; H2: preterm birth is associated with low educational attainment; H3: school dropout is associated with early marriage. Hypothesis‐specific search terms and eligibility criteria were applied. We also tested hypothesis H4: preterm birth is associated with reduced cognitive function, by evaluating systematic reviews of research from any setting.

**Results:**

We identified 184 empirical articles for H1–H3, with 26 satisfying the criteria for full review, and 5 systematic reviews for H4. The available evidence supported H1 and H3, but was weak for H2. For H3, studies indicated contrasting directions of association. The systematic reviews demonstrated evidence supporting H4. The majority of empirical studies reviewed had a low risk of bias.

**Conclusions:**

An intergenerational cycle of risk linking early marriage, preterm delivery and low educational attainment is plausible, involving both behavioral pathways (e.g., school dropout and early marriage) and biological mechanisms (e.g., preterm birth and cognitive function). Few studies have investigated the prospective associations of preterm birth with school outcomes, or school dropout with early marriage, in low‐ and middle‐income countries.

## Graphical abstract

We conducted systematic searches to review evidence from low and middle‐income countries for an intergenerational cycle of risk, linking early marriage, preterm delivery and low educational attainment. The cycle is plausible, involving both behavioural and biological pathways, however few studies in these settings have investigated educational outcomes following preterm birth.

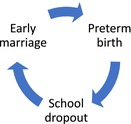

## 1 Introduction

Early childbearing is detrimental to maternal and child health (Fall et al. 2015; Marphatia et al. 2017), but is positively correlated with fertility (Raj et al. 2009; Sagalova et al. 2021). From an evolutionary perspective, this indicates a trade‐off between life‐history traits, whereby increased allocation of resources to reproduction in women comes at the expense of their investment in growth and maintenance (Marphatia, Saville, Manandhar, Cortina‐Borja, Reid, and Wells 2021). This trade‐off may also be subject to sexual conflict over the timing of the onset of reproduction, as fathers gain the fitness benefits of women reproducing early, without themselves paying the health costs (J. C. Wells 2022).

In many countries, particularly in South Asia, early age of reproduction is preceded by high rates of early marriage, as reproduction outside marriage is socially unacceptable (Marphatia et al. 2017). In other settings, early marriage may be the socially driven consequence of an early pregnancy. According to data from 2015 to 2024, 19% of women aged 20–24 years across 121 countries were married by age 18 years and 4% by the age of 15 years (UNICEF 2025). Globally, the prevalence of child marriage has decreased moderately from 21.9% in 2015 to 18.6% in 2025 (Child Marriage Data Portal 2025). Central and Southern Asia have made the most progress, from 37.3% to 25% between 2015 and 2025, whereas the decrease in sub‐Saharan Africa is much lower, from 38.2% to 30.8%.

Early marriage recurs across generations in high‐risk groups (Marphatia, Wells, Reid, Bhalerao, and Yajnik 2025), and may be part of a broader intergenerational cycle of disadvantage that embraces poverty, malnutrition, gender inequality, early reproduction, and risky behavior (J. C. Wells et al. 2019). Early marriage has detrimental effects on women beyond early reproduction, and is associated with a range of adverse outcomes including lower schooling, low autonomy, and intimate partner violence (Kidman 2017; Marphatia et al. 2017; Subramanee et al. 2022). It is therefore the target of extensive policy efforts (Malhotra and Elnakib 2021b; UNICEF 2023).

More broadly, intergenerational associations have been documented for a range of adverse socioeconomic and human capital outcomes. For example, low parental education predicts poorer schooling outcomes in the offspring (Marphatia et al. 2016, 2019), while poverty also tracks across generations (Harper et al. 2003; Van Ryzin et al. 2018). In the 1960s, Oscar Lewis presented a “culture of poverty” hypothesis, proposing that the perpetuation of adverse socioeconomic outcomes across generations was partly driven by the transmission of cultural values (Lewis 1966). In the US, this theory stimulated a “war on poverty” with the aim of “correcting” such values. When the programs, which made little effect to change structural factors, did not succeed, the intractability of the “culture of poverty” was invoked as the explanation—its intergenerational “cultural basis” had seemingly made poverty ineradicable (Seligman 1968).

Although Lewis encouraged policy makers to view behaviors as learned and culturally transmitted, they might also be sensitive to physiological mechanisms and biological exposures. Among the traits that Lewis considered characteristic of the culture of poverty are several that are highlighted in evolutionary life‐history theory, including low life expectancy, early initiation into sex, and high mortality risk (Lewis 1966; Promislow and Harvey 1990). Over recent decades, the notion of behavior and physiology interacting dynamically across intergenerational timescales has attracted increasing attention. For example, there is growing interest in how psychosocial stressors and material inequalities “get under the skin” and impair health and development across generations (Cheng et al. 2016; Entringer et al. 2011; Yehuda et al. 2016). Exposure to pollutants and toxins may exert similar effects (Lowell et al. 2022; Schell 1997).

For early marriage, the primary driving factor is widely assumed to be household poverty (Psaki et al. 2021), and this has led to cash‐transfer programs aiming to delay girls' marriage. These programs have generally had low efficacy (Malhotra and Elnakib 2021a). However, a major limitation of many studies that linked early marriage with poverty is that economic assets were measured only in households where the woman was already married, thus indexing marital rather than natal household wealth (Marphatia, Saville, Manandhar, Cortina‐Borja, Wells, and Reid 2021). In a rare study where assets were measured in the natal household, girls' low education was a robust predictor of age at marriage, whereas independent of that, household assets showed no association (Marphatia, Saville, Manandhar, Cortina‐Borja, Wells, and Reid 2021).

In South Asian societies, marriage decisions are also strongly influenced by sociocultural norms relating to a range of factors, including cementing family ties, dowry payments, ensuring brides' chastity, and subservience (Fattah and Camellia 2022; UNFPA 2019). In India, sociocultural norms for marriage age are changing more slowly than those for education (Marphatia, Wells, Reid, et al. 2025). However, if marriage decisions are considered entirely the product of such norms, then difficulties in changing marriage practices might appear to be reiterating the “culture of poverty” argument—that early marriage is a cultural practice that is simply “too engrained” to change.

Here, we draw on emerging evidence linking early women's marriage with the risk of adverse physical outcomes in the offspring, to generate a new overarching hypothesis: that the perpetuation of early marriage across generations involves the interaction of behavioral decisions with physiological traits, contributing to a complex multi‐mechanism intergenerational cycle of risk. By specifying and addressing the consecutive risks, we might be able to reduce the intergenerational propagation of disadvantage. Our recent research in lowland rural Nepal found that independent of age at first childbearing, girls' early marriage was associated with an increased risk of delivering a preterm infant (Miller et al. 2022). In the same cohort, we had also shown that girls' school dropout was associated with their early marriage (Marphatia et al. 2020). Like early marriage, preterm birth is a global health concern, affecting 12.5% of births in low‐income countries and 8.8% in middle‐income countries (Swarray‐Deen et al. 2024). There has been very little progress in reducing preterm birth globally, from 13.8 million in 2010 to 13.4 million in 2020 (Ohuma et al. 2023). Risk factors include several that relate to early marriage, including maternal age < 20 years, lack of education and nulliparity (Swarray‐Deen et al. 2024).

Given that in a high‐income setting, shorter gestation length showed a dose–response association with difficulties in school (MacKay et al. 2010), it seems possible that early marriage, preterm birth, and poor schooling might reinforce each other across generations, and hence form one component of the broader intergenerational cycle of disadvantage. From an evolutionary life‐history perspective, exposures that undermine maternal investment in daughters' longevity may favor an earlier shift of daughters' metabolic resources to reproduction (Griskevicius et al. 2011; J. C. Wells et al. 2019). In contemporary human settings, such interactions may be mediated by both physiological mechanisms and cultural institutions such as school dropout and early marriage.

**FIGURE 1:**
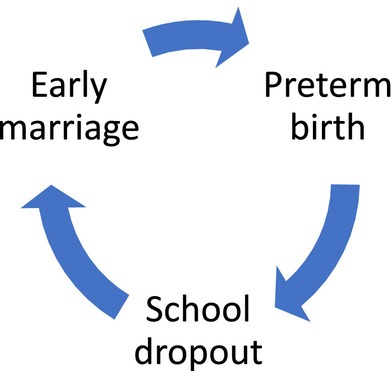
Schematic diagram of the hypothesized intergenerational cycle, linking girls' early marriage, preterm birth, and school dropout. The associations are not deterministic at the individual level; rather at the population level, each outcome in the cycle increases the probability of the next outcome occurring.

This component of the cycle of disadvantage is therefore projected to incorporate three specific risks, framed here as hypotheses (H) and illustrated in Figure 1:

*Girls' early marriage is associated with increased risk of delivering a preterm infant*.

*Preterm birth compromises educational attainment of the offspring, increasing the risk of school dropout*.

*Poor educational attainment and school dropout increase the likelihood of girls' early marriage*.

Whether epidemiological evidence supports these hypotheses in low‐ and middle‐income countries (LMICs), where early marriage is especially common (Marphatia et al. 2017), remains unclear. We therefore conducted systematic literature reviews to test these hypotheses. As prospective longitudinal studies of cognitive function in school children in LMICs remain sparse, we also searched for systematic reviews of research from any setting, to test a further hypothesis relating to underlying mechanisms:

*Preterm birth is associated with reduced cognitive function in school‐aged children*.

## 2 Methods

Given the complexity of our overarching hypothesis, we conducted a systematized review (Grant and Booth 2009), comprising four systematic searches with specific eligibility criteria. This enabled us to integrate the findings of these searches into a broader synthesis.

### 2.1 Search Strategy

The literature searches were conducted using the electronic databases Scopus, PubMed, and Google Scholar in March 2025, considering studies published in from 2000 to 2025 in English. For each hypothesis, broad search terms were applied to both titles and abstracts to increase the likelihood of identifying relevant articles. The search terms were as follows:

*Early Marriage and Preterm Birth: (“Early marriage” or “child marriage” or “married girls” or “adolescent marriage” or “child and adolescent marriage” or “married adolescent” or “underage marriage” (and not “adolescent pregnancy” or “early childbearing”)) and (“preterm birth” or “preterm delivery” or “premature birth”)*.

*Preterm Birth and Educational Attainment: (“Preterm birth” or “preterm delivery” or “premature birth”) and (“schooling” or “school dropout” or “education” or “educational attainment” or “educational status” or “educational level” or “educational achievement” or “educational outcome” or “educational disadvantage” or “age at school entry” or “school grade” or “school level” or “school performance” or “schooling attainment”)*.

*Educational Attainment and Early Marriage: (“schooling” or “school dropout” or “education” or “educational attainment” or “educational status” or “educational level” or “educational achievement” or “educational outcome” or “educational disadvantage” or “age at school entry” or “school grade” or “school level” or “school performance” or “schooling attainment”) and (“early marriage” or “child marriage” or “married girls” or “adolescent marriage” or “child and adolescent marriage” or “married adolescent” or “underage marriage” and not (“adolescent pregnancy” or “early childbearing”))*.

*H4 included the same search terms as H2 but searched for systematic reviews of studies in any setting. Broader school and education search terms were retained as research on school performance and educational attainment frequently includes approaches and scales reflecting cognitive skills and development (such as literacy and intelligence quotient). The inclusion criteria differed from H2*, *however, as detailed in the subsequent section*.

### 2.2 Inclusion and Exclusion Criteria

For [Link], [Link], [Link], only empirical studies conducted in LMICs with a quantitative design were eligible. We included cross‐sectional, cohort and case–control studies and randomized controlled trials. Studies using retrospective self‐report data on preterm birth, length of school attendance/school dropout and age at marriage were included, though for preterm birth, parents were reporting the original clinical categorization. Qualitative studies were excluded. If mixed‐method studies were identified, only quantitative findings were analyzed.

For H1, articles were only included if they investigated the association between early marriage and preterm birth. The population of interest was girls or women living in LMICs, the exposure of interest was early marriage, and the outcome of interest was preterm birth in the girls' or women's offspring. The comparison group consisted of women who had not married early. Studies with no comparison group were excluded. Early marriage and preterm birth were defined as described in the definitions section below.

For H2, articles were only included if they investigated the association between preterm birth and educational attainment/school performance/school dropout. The population of interest was school‐aged children living in LMICs, the exposure of interest was preterm birth, and the outcome of interest was school attainment, performance or dropout. The comparison group consisted of children who were not born preterm. Studies with no comparison group were excluded. Preterm birth was defined as described in the definitions section below.

For H3, articles were only included if they investigated the associations between educational attainment/school performance/school dropout and early marriage. The population of interest was girls/women living in LMICs, the exposure of interest was school attainment, performance or dropout, and the outcome of interest was early marriage. The comparison group consisted of women with better educational outcomes (e.g., higher attainment, performance or not having dropped out). Studies with no comparison group were excluded.

For H4, only systematic reviews of quantitative studies (as defined for [Link], [Link], [Link]) were included, with no restriction on country in the search operation. The population of interest was school‐aged children, the exposure of interest was preterm birth, and the outcome of interest was cognitive function.

For [Link], [Link], [Link], [Link] only published peer‐reviewed full research articles were included, abstracts with no associated full research article were excluded. Gray literature was also excluded. Articles were excluded if they discussed genetic traits as predisposing factors, focused on early marriage in boys, and/or focused on early childbearing without marriage.

Educational attainment was defined as the number of years completed in school, while school dropout was defined as leaving school prior to the completion of specified curriculum requirements. No strict definition was applied to define school performance, as a wide range of assessments exists worldwide. Early marriage was defined as a formal or informal union of a girl‐child under 18 years old, with no restriction on the age of the spouse (UNICEF 2018).

Preterm birth was defined as a live birth below 37 weeks of gestation (Blencowe et al. 2012). Any method of assessment of gestational age was accepted. For H1 and H2, given limited evidence, no restriction was applied to the degree of preterm delivery (extremely [< 28 weeks], very [28 to < 32 weeks], and late preterm [32–37 weeks]; WHO 2023).

LMICs were categorized using the 2022 World Bank definition of countries with gross national income under $13 205 (World Bank 2022). All countries within the low, lower‐middle, and upper‐middle groups fell into this category.

### 2.3 Data Extraction and Calculations

For each hypothesis, results from different databases were collated in Endnote, where duplicates were removed using automated functions and manual methods. Titles and abstracts were assessed for relevance. Full texts were then checked for inclusion and exclusion criteria. These steps were conducted by ER with JW acting as the second reviewer. Any discrepancies in the selected papers were discussed with co‐authors.

Once a study was considered to satisfy eligibility criteria, it was subjected to analysis and the findings were summarized. For [Link], [Link], [Link], data were extracted for the following variables: sample size, study design, demographic characteristics, country, operational definition for exposure and outcome, adjusted factors, and main findings. For H4, data were extracted for the following variables: number of studies, setting, operational definition for exposure and outcome, risk of bias assessment, and main findings.

Two of the studies for H1 (Urquia, Batista, Grandi, et al. 2022; Urquia, Batista, Cunha Cardoso, et al. 2022) contained data that allowed quantification of the risk of preterm birth associated with early marriage, but did not report the results directly. We therefore calculated the risk as follows. We used women married 20–24 years (*M*) as the reference group, and evaluated the risk of preterm birth (*P*) compared to term birth (*T*) for women married at earlier ages (EM). We calculated crude odds ratios (OR) from numbers (*n*) as follows:

$$\text{OR}=\left[\left(n-P-\mathrm{EM}\right)/\left(n-P-M\right)/\left(n-T-\mathrm{EM}\right)/\left(n-T-M\right)\right]$$

### 2.4 Risk of Bias Assessment

For [Link], [Link], [Link], a risk of bias assessment was carried out for each study using the Newcastle–Ottawa Scale for case control and cohort studies (G. A. Wells et al. 2000) or a modified version of this for cross‐sectional studies (Carra et al. 2025). The questions used are given in Supporting Information. This assessment considers the possible level of bias of the study through criteria reflecting sample selection, comparability of outcome categories or cases and controls, quality of variable assessments, and/or loss to follow‐up (for longitudinal studies). A score of up to 9 points is given, with higher scores indicating a lower risk of bias. The scores are provided alongside the extracted data for each study and the main reasons for the given scores are summarized in the results. Studies were considered to have low, moderate or high risk of bias if they had scores of 7–9, 4–6, or 1–3 respectively (Carra et al. 2025).

## 3 Results

Across hypotheses 1 to 3, a total of 184 empirical articles were found and 26 articles satisfied the criteria for full review.

### 3.1 Study Characteristics

For H1, there were four studies: one longitudinal study from Nepal, and three cross‐sectional studies from India, Brazil, and Ecuador. The studies from Brazil and Ecuador were national studies covering all live births to women below 25 years in 2011–2018 (Brazil) and 2014–2018 (Ecuador) (Urquia, Batista, Grandi, et al. 2022; Urquia, Batista, Cunha Cardoso, et al. 2022). One of these studies also included data from the USA and Canada but these are not reported here. The study from India was a small study of 158 married couples in a village in Gujarat, India (Pandya and Bhanderi 2015). The study from Nepal assessed a sample of close to 18 000 married girls/women aged 10–49 years from a trial conducted in rural lowland Nepal (Miller et al. 2022). These studies all explored associations of early marriage (either defined as marriage below 18 years, or at specific ages below 18, or using marital status alongside maternal age) with preterm birth. One study did not define preterm birth by gestational age (Pandya and Bhanderi 2015), and one study distinguished subtypes of preterm status (Urquia, Batista, Cunha Cardoso, et al. 2022).

Three of the studies were found to have a low risk of bias with scores of 7 (Miller et al. 2022) and 8 (Urquia, Batista, Cunha Cardoso, et al. 2022; Urquia, Batista, Grandi, et al. 2022). Risk of bias arose from reliance on self‐report for assessments of preterm birth and early marriage (Miller et al. 2022; Urquia, Batista, Cunha Cardoso, et al. 2022; Urquia, Batista, Grandi, et al. 2022) and high loss to follow‐up (Miller et al. 2022). The other study had a score of 0 indicating a possible very high risk of bias (Pandya and Bhanderi 2015). The main reasons were that none of the variables were described in the methods and confounding was not considered.

For H2, only one study was identified, assessing associations of preterm birth with educational attainment in 4518 adults across five countries (Brazil, Guatemala, India, the Philippines, South Africa) (Stein et al. 2013). This study had a low risk of bias (score of 7). Points were lost due to preterm birth assessment relying on self‐report and because while some adjustments were made in the analyses for sex, age and study site, others like socioeconomic and ethnic background were not considered.

For H3, 20 studies were identified analyzing data from 31 countries, spanning sub‐Saharan Africa (25 countries) (Bengesai et al. 2021; Bhan et al. 2019; Fang et al. 2024; Glick et al. 2015; Glynn et al. 2018; Lami et al. 2023; Sagalova et al. 2021; Zegeye et al. 2021), Asia (5 countries) (Bhan et al. 2019; Cameron et al. 2023; Kanji et al. 2024; Kumar et al. 2023; Y. Liang and Yu 2022; Marphatia et al. 2020; Marphatia, Wells, et al. 2021; Paul 2019, 2020; Prakash et al. 2017; Roy and Chouhan 2021; Sekine and Hodgkin 2017; Singh et al. 2024), and South America (1 country) (Bhan et al. 2019). Twelve analyses were cross‐sectional and eight had a longitudinal design. Not every country was analyzed independently; for example one study pooled data from 21 sub‐Saharan African countries (Sagalova et al. 2021), while another study pooled data from India, Ethiopia, Vietnam, and Peru (Bhan et al. 2019). The most commonly studied country was India (nine studies). Fifteen studies investigated only educational attainment (Bengesai et al. 2021; Cameron et al. 2023; Fang et al. 2024; Glick et al. 2015; Kanji et al. 2024; Lami et al. 2023; Y. Liang and Yu 2022; Marphatia et al. 2020; Marphatia, Wells, et al. 2021; Paul 2019, 2020; Roy and Chouhan 2021; Sagalova et al. 2021; Singh et al. 2024; Zegeye et al. 2021), four studies discussed mainly school dropout (Bhan et al. 2019; Kumar et al. 2023; Prakash et al. 2017; Sekine and Hodgkin 2017), and one study investigated both educational attainment and school performance (using age for grade) and school dropout (Glynn et al. 2018). Ten studies described EM as the exposure, of which 4 were longitudinal (Bhan et al. 2019; Cameron et al. 2023; Kanji et al. 2024; Kumar et al. 2023), and 10 described EM as the outcome, of which 4 were longitudinal (Glynn et al. 2018; Y. Liang and Yu 2022; Marphatia et al. 2020; Marphatia, Saville, Manandhar, Cortina‐Borja, Reid, and Wells 2021; Marphatia, Saville, Manandhar, Cortina‐Borja, Wells, and Reid 2021; Marphatia, Wells, et al. 2021).

For the most part, the risk of bias in H3 studies was low to moderate with five studies having low scores of 3 or 4, one study a score of 5 and all other studies scores of 6 or 7. Of the eight longitudinal studies, all had low risk of bias. Scores were lost mainly due to having self‐reported measures (all studies), not adjusting for confounding or not describing this in the methods (Kumar et al. 2023; Marphatia et al. 2020), not describing loss to follow‐up (all studies except Kumar et al. 2023; Marphatia et al. 2020), or having samples that were not representative of the population of interest (i.e., all subjects used or random‐sampling and appropriate weighting used) (Marphatia et al. 2020). Among the cross‐sectional studies with scores of 5–7, points were lost for similar reasons, including measures being self‐reported (all studies), samples not being representative of the population of interest (Glick et al. 2015) and confounders not adequately described and/or controlled for (Prakash et al. 2017; Sagalova et al. 2021; Sekine and Hodgkin 2017). Other studies with lower scores were studies that, in addition to any other issues described previously, assessed risk factors for early marriage by including all risk factors together with no consideration for confounding nor discussion of causality (Paul 2020; Roy and Chouhan 2021; Singh et al. 2024; Zegeye et al. 2021).

For [Link], [Link], [Link], the sample size varied from 158 to 7 953 376, since many studies used national demographic or health survey data. For H4, five systematic reviews were identified, summarizing the results of a total of 217 original studies.

## 4 Main Findings

### 4.1 H1: Girls' Early Marriage Is Associated With an Increased Risk of Delivering a Preterm Infant

Table 1 summarizes the study characteristics and main findings for the studies on early marriage and preterm birth (H1) (Miller et al. 2022; Pandya and Bhanderi 2015; Urquia, Batista, Cunha Cardoso, et al. 2022; Urquia, Batista, Grandi, et al. 2022). All four studies reported an association of early age at reproduction with an increased risk of having a preterm delivery, though the magnitude of effect varied by setting, and one study found that the effect was only significant in primigravidae. The two South American studies focused on age at reproduction as the primary exposure; however the reported data allowed calculation of the associations for early marriage. In both Ecuador and Brazil, earlier marriage was associated with increased odds of preterm birth, with the earlier the marriage, the greater the risk. In the two South Asian studies, where marriage is a near‐universal precedent of childbearing, earlier age at marriage was again associated with increased risk of preterm delivery in a dose–response manner. In the study from Nepal, the association was only apparent in first‐time mothers, but was independent of the age at childbearing.

**TABLE 1:** Summary of studies on early marriage and preterm birth.

Author (year)	Country	Study design	Sample	Exposure outcome(s)	Adjusted factors	Findings	RoB score
Miller et al. (2022)	Nepal	Longitudinal (secondary analyses of randomized‐control trial data)	17 974 married and conceived women aged 10–49 years	EM: < 14, 15, and 16–17 years PB: < 37 weeks/259 days	Maternal education, caste group, material assets, maternal nutritional status (BMI, MUAC, Height)	Primigravidae married < 14 years greater likelihood of PB (OR: 1.28 [CI: 1.01, 1.62]); no significant result for girls married at 15 years (1.12 [0.89, 1.41]) or 16–17 years (1.12 [0.90, 1.39]). No significant association in multigravidae	7
Pandya and Bhanderi (2015)	India	Cross‐sectional	158 couples	EM: < 18 years from self‐report/marriage certificate PB: Not defined. Based on records such as Mamta Card or self‐response	None	Prevalence of PB higher in women married < 18 years compared to married 18+ years (OR: 4.74 [CI: 1.01, 22.36]^a^). No further analyses carried out	0
Urquia, Batista, Cunha Cardoso, et al. (2022)	Brazil	Cross‐sectional	7 953 739 births to mothers < 25 years	EM: 16–17 and ≤ 15 years PB: very preterm (24–31 weeks) and moderately preterm (32–36 weeks)	Father's age, late prenatal care initiation, maternal education, year of birth, infant sex	Compared to married 20–24 year old women: women/girls married at 18–19, 16–17 and < 16 years had higher odds of very PB (OR [CI]: 1.09 [1.04, 1.15], 1.44 [1.33, 1.56], 2.13 [1.71, 2.67])^a^; and women/girls married at 18–19, 16–17 and < 16 years had higher odds of moderate PB (OR [CI]: 1.12 [1.10, 1.14], 1.39 [1.36, 1.43], 1.76 [1.63, 1.91])^a^	8
Urquia, Batista, Grandi, et al. (2022)	Brazil, Ecuador (USA, Canada but not included here)	Cross‐sectional	Brazil: 24 446 170 births, Ecuador: 1 581 550 births to mothers < 25 years	EM: assessed from marital status and maternal age at the time of birth PB: < 37 weeks gestation	Infant sex, previous birth, year of birth, maternal ethnicity Brazil only: paternal age, prenatal care initiated in 1st trimester, state, age‐appropriate low education Ecuador only: foreign‐born mother, adequacy of the number of prenatal care visits for gestational age (WHO), maternal literacy, maternal region of residence and rurality	In Brazil, compared to married 20–24 year old women, women/girls married at 18–19 and < 18 years had higher odds of PB (OR [CI]: 1.12 [1.10, 1.14] and 1.43 [1.40, 1.47] respectively)^a^ In Ecuador, compared to married 20–24 year old women, women/girls married at 18–19 and < 18 years had higher odds of PB (OR [CI]: 1.08 [1.00, 1.17] and 1.13 [0.98, 1.30] respectively)^a^	8

Abbreviations: BMI, body mass index; CI, confidence interval; EM, early marriage; MUAC, mid‐upper arm circumference; OR, odds ratio; PB, preterm birth; RoB, risk of bias.

^a^ Crude OR and 95% CI estimated from crude numbers reported in the study.

### 4.2 H2: Preterm Birth Compromises Educational Attainment of the Offspring

Table 2 summarizes the study characteristics and main findings for the association of preterm birth and educational attainment/academic performance/school dropouts (H2). A pooled analysis of five birth cohorts from Guatemala, Brazil, South Africa, India, and the Philippines found that compared with adults born at term, those born preterm had 0.44 years lower (95% CI, 0.17–0.71 years) educational attainment. However, there was heterogeneity across countries with stronger negative associations only found in India and the Philippines and other countries showing little/no evidence of any association (Table 2).

**TABLE 2:** Summary of studies on preterm birth and educational outcomes.

Author (year)	Country	Study design	Sample	Exposure outcome(s)	Adjusted factors	Findings	RoB score
Stein et al. (2013)	Brazil, Guatemala, India, the Philippines, and South Africa	Prospective cohort	4518 adults	PB: < 37 completed weeks of gestation Educational attainment: completed years of schooling	Site, sex, and age at adult assessment	Compared with adults born term, educational attainment was 0.44 years lower (CI, 0.17, 0.71 years) in those with PB. However, this varied across countries (*p* < 0.1) with PB associated with fewer years of school in India (−1.14 [−1.73], −0.56) and the Philippines (−0.43 [−0.84, −0.01]) but less so in Brazil (−0.06 [−1.01, 0.89]) and South Africa (−0.05 [−0.39, 0.28]) and not at all in Guatemala (0.88 [−0.47, 2.24]). No differences reported by sex	7

Abbreviations: CI, confidence interval; OR, odds ratio; PB, preterm birth; RoB, risk of bias.

### 4.3 H3: Poor Educational Attainment Increases the Likelihood of Girls' Early Marriage

Table 3 summarizes the study characteristics and main findings for the studies on educational attainment, academic performance or school dropout and early marriage (Bengesai et al. 2021; Bhan et al. 2019; Cameron et al. 2023; Fang et al. 2024; Glick et al. 2015; Glynn et al. 2018; Kanji et al. 2024; Kumar et al. 2023; Lami et al. 2023; Y. Liang and Yu 2022; Marphatia et al. 2020; Marphatia, Wells, et al. 2021; Paul 2019, 2020; Prakash et al. 2017; Roy and Chouhan 2021; Sagalova et al. 2021; Sekine and Hodgkin 2017; Singh et al. 2024; Zegeye et al. 2021). All 20 studies reported an association between lower education and early marriage, though the nature of the association varied across studies and the direction of the association was analyzed in different ways. All 15 studies that tested whether educational attainment was lower among early‐married girls, or whether increased education was associated with reduced odds of early marriage, supported the hypothesis. All four studies that investigated whether early‐married girls were more likely to have dropped out of school, or whether school dropout was associated with increased odds of early marriage, supported the hypothesis. The available evidence indicates that the association of early marriage with poor education may be a two‐way street, with each a risk factor for the other.

**TABLE 3:** Summary of studies on educational outcomes and early marriage.

Author (year)	Country	Study design	Sample	Exposure outcome(s)	Adjusted factors	Findings	Bias score
Bengesai et al. (2021)	Zimbabwe	Cross‐sectional	2380 ever‐married women aged 20–29 years	EM: Age at marriage < 18 years Lower secondary school completion (11 years): Yes, no	Place of residence, religion, spouse education (years), type of marital union (monogamy/polygamy/missing), age at sexual debut (including informal union), sex of HH head, spousal age difference, age of respondent	Prevalence of lower secondary school completion 62% lower in EM women (PR [CI]: 0.381 [0.30, 0.49]) compared to those married later (≥ 18 years)	7
Bhan et al. (2019)	India, Ethiopia, Vietnam, Peru	Prospective cohort	1338 girls	EM: Age at marriage < 18 years School dropout: dropped out by age 12/15 years versus stayed in school	Country, wealth quartiles, rural/urban residence, maternal education (none, primary, secondary or higher), whether parents are alive, early menarche, parental beliefs on value of education	Girls married before 16 years, at 16–17 years and 18–19 years had a greater risk of school dropout by age 12/15 years compared to girls not married by 19 years (RRR [CI]: 11.76 [6.97, 19.8], 4.91 [3.07, 7.86], 4.86 [2.79, 8.46] respectively). Note that no girls married at ages 16–17 and 18–19 years in Ethiopia and it is therefore not included in those results	7
Cameron et al. (2023)	Indonesia	Prospective cohort	40 800 men and women (only women reported here)	EM: Age at marriage < 18 years Educational attainment: total years completed by the time of the survey	#A Urban area, Muslim, age, parental education, parent marital status (at age 12 years), and village fixed effects (accounting for differences between individuals in the same villages) #B Urban area, age, and sister fixed effects (controls for all family characteristics, both observable and unobservable, experienced by sisters, e.g., family background)	Women who marry before the age of 18 obtained 1.65 years (SD: 0.09) less education after adjusting for #A and 0.91 years (SD: 0.22) less education after adjusting for #B	7
Fang et al. (2024)	Nigeria	Cross‐sectional	Women aged 15–29 years in 42 000 households	EM: Age at first marriage < 18 years Educational attainment: completed secondary education or attained a higher level of education	Women's age in 5‐year groups, de facto region of residence, area of residence (urban, rural), current marital status of the respondent, and family poverty (living in households in the lowest wealth quintile)	The marginal effect of child marriage on whether the respondent completed secondary or higher education was −22.98%	7
Glick et al. (2015)	Madagascar	Cross‐sectional	2336 females aged 12–25 years	Educational attainment: number of years completed EM: Age at marriage	Year of birth, family background (parent education, parent mortality, assets, religion, HH head ethnicity), and community variables (province indicators, urban residence, electricity, water source, distance to public services, transport, and infrastructure)	Each additional year of education associated with 1.5 year increase median marriage age	6
Glynn et al. (2018)	Malawi	Prospective cohort	8576 girls aged over 12	Educational attainment: years overage for grade School dropout: in primary school or beyond EM: Age at first marriage	Parental education, vital status of parents, living arrangements (HH size, number of children under 5 in HH, living with parents), sex of head HH, SES, year of interview	Compared to girls in primary school, rates of marriage across ages 13 to 18 years were cumulatively higher for girls who had dropped out in primary school (e.g., marriage by 13 years HR 1.79 [CI 1.20, 2.66], marriage by 16 years HR 4.67 [CI 2.98, 7.32]). Similar results for those who dropped out of school beyond primary (e.g., marriage by 15 years HR 2.30 [CI 1.07, 4.92], marriage by 18 years HR 3.58 [CI 2.17, 5.90])	6
Kanji et al. (2024)	India	Prospective cohort	1008 children aged 8 up to 22 years	EM: Age at marriage < 18 years Educational attainment: highest school grade attained at different ages	None	Lower mean educational attainment found at age 12 years (but not 8 years) for girls married before 18 years compared to girls not. Difference in difference analyses show EM negatively affects women's self‐reported health and educational attainment by age 22 years (from baseline 8 years)	7
Kumar et al. (2023)	India	Prospective cohort/panel	6178 adolescent girls enrolled in school at survey wave 1	EM: Married at age 15–19 years School dropout: dropped out of school between survey waves 1 and 2	None	Prevalence of school dropout higher in married than unmarried girls at 84.3% versus 45.8% (no further statistical analyses carried out)	6
Lami et al. (2023)	Ethiopia	Cross‐sectional	990 women of reproductive age	EM: Age at marriage < 18 years Educational attainment: no formal education, primary school, secondary school, or diploma and above	Age, religion, husband's educational level, occupation of women, husband's occupation, residence, means of engagement/marriage, consent at marriage, knowing the age of legal marriage, and decision‐maker at marriage	Women who had a diploma and above educational level were 74% [AOR: 0.26 (0.10, 0.70)] less likely to have had a child marriage than those who had a primary school educational level (reference group). No associations were found for other education levels (no formal education, primary school, secondary school)	3
Liang and Yu (2022)	China	Panel	14 218 HH	Educational attainment: Years of schooling completed EM: Age at first marriage (< 16, < 18 or < 20/22 years)	Parental years of schooling, parental age, gender, ethnic group, urban registration, migration status, parental absence in mid childhood, birth cohort, region, province	A 1‐year increase in schooling was associated with a lower likelihood of marriage before 16 and 18 years (coeff: −0.01 [SE: 0.01; *p* < 0.01] and −0.02 [0.01; *p* < 0.05] respectively) but not marriage before 20/22 years (0.02 [0.02])	7
Marphatia, Wells, et al. (2021)	India	Prospective cohort	648 mother and child pairs	Educational attainment: completion of lower secondary education (10th standard) EM: Age at marriage < 19 years	Agrarian landholding, maternal parity, caste, nuclear household, low paternal education, lower gestation, and girls' poor infant growth, measured prospectively in the natal household in early life	Girls who had not completed lower secondary school had 9.20 the odds of early marriage compared to those who did (AOR: 9.20 [95% CI: 2.78, 30.44])	7
Marphatia et al. (2020)	Nepal	Longitudinal (secondary analyses of randomized‐control trial data)	6406 women aged 23–30 years	Educational attainment: none, 1–5 (primary), 6–8 (lower secondary), 9–10 (secondary), or 11–13 years (higher secondary or above) EM: Age at marriage ≤ 15 years (childhood), 16–17 years (adolescence) or ≥ 18 years	Husband's education, caste	Women's later greater educational attainment was associated with age at marriage ≥ 18 years, though most strongly with 8 or more years of education (OR 1.03 [95% CI: 0.83, 1.28], 1.27 [0.96–1.65], 2.22 [1.70–2.90], 5.02 [3.48–7.25] for 1–5 years, 6–8 years, 9–10 years and 11–13 years of education respectively versus none [reference group])	7
Paul (2019)	India	Cross‐sectional	122 955 women aged 20–24 years in 640 districts	Educational attainment: No education/illiterate, primary (1–5 years), secondary (5–10 years), higher secondary (10–12 years) college/higher (12+ years) EM: Age at first marriage < 18 years	Urbanization, religion (Hindu population), women autonomy (HH decision making, physical mobility, access to economic resources), and region	Districts with a higher percentage of girls with no education/illiteracy and primary education had an increased risk of EM (coeff: 0.52 [SE: 0.03] and 0.66 [0.11] respectively with *p* < 0.01) Districts with a higher percentage of girls with secondary, higher secondary, and college/higher education had a reduced risk of EM (−0.31 [0.06], −1.49 [0.09], and −1.31 [0.08] respectively with *p* < 0.01 or 0.05)	6
Paul (2020)	India	Cross‐sectional	122 955 women aged 20–24 years	Education level: Illiterate, primary, secondary, higher EM: Age at first marriage < 18 years	Socio‐economic characteristics of women (i.e., place of residence, caste, religion, education and wealth quintile)	Compared to illiterate women, the odds of EM decreased with an increase in education level: Primary school (OR: 0.87 [CI: 0.83, 0.92]) Secondary school (0.53 [0.50, 0.55]) Higher school (0.17 [0.16, 0.19])	4
Prakash et al. (2017)	India	Cross‐sectional	2275 adolescent girls aged 13–14	EM: Engaged/married School dropout: currently in school (yes/no) School absenteeism: Absent from school for 4+ days in past month were categorized as frequently absent	Cohort wave and district, age caste, female headed households, family wealth, family debt, family support for girls' education, number of siblings, older/younger sibling.	EM girls had higher odds of dropping out of school compared to never married girls (OR: 3.69 [CI: 2.12, 6.40])	6
Roy and Chouhan (2021)	India	Cross‐sectional	357 women aged 15–49 years	Education level: Illiterate, primary, secondary, higher EM: Age at marriage < 18 years	Age, socioeconomic variables including religion, social group, parental education, father occupation, family income	Compared to illiterate women, the odds of EM decreased with an increase in education level: Primary school (OR: 0.70 [CI: 0.32, 0.98]) Secondary school (0.72 [0.36, 0.81]) Higher school (0.06 [0.02, 0.19])	4
Sagalova et al. (2021)	West and Central Africa (WCA)	Cross‐sectional	262 721 girls aged 15–19 years	EM: Age at marriage 10–14 years or 15–19 years Educational attainment: None/< primary, primary, incomplete secondary, secondary, or higher	Cultural norms, average living standards	Across WCA, first marriage at age 10–14 years and ages 15–19 years associated with a higher likelihood of having educational attainment below primary school (coeff: 0.29 [SE: 0.003; *p* < 0.001] and 0.20 [0.002]; *p* < 0.001)	5
Sekine and Hodgkin (2017)	Nepal	Cross‐sectional	1631 girls aged 15–17 years	EM: Age at first marriage School attendance: Yes, no School dropout < secondary school (17 years): Yes, no	Age, urban/rural residence, religion, HH wealth, social class, HH head education	Married girls had lower odds of school attendance (OR: 0.10 [CI: 0.06, 0.17]; *p* < 0.001) and dropout (10.04 [5.84, 17.25]; *p* < 0.001) compared to those unmarried	6
Singh et al. (2024)	India	Cross‐sectional	171 199 ever or currently married women aged 20–29 years	Educational status: No education, Primary, Secondary, and Higher EM: Age at first marriage < 18 years	Individual‐level factors such as age, education, mass media exposure, and prior relationship with the husband, household‐level factors (not specified) and household/community‐level factors (not specified)	As education increases, the likelihood of early marriage decreases significantly. Women with primary education (OR: 0.93 [CI: 0.89, 0.97]), secondary education (0.53 [CI: 0.51, 0.54]), and higher education (0.12 [CI: 0.11, 0.12]) all have lower odds of early marriage compared to those with no formal education	4
Zegeye et al. (2021)	Mali	Cross‐sectional	8350 women aged 18–49 years	Educational level: No formal education, primary school, secondary school, higher EM: Age at marriage < 18 years	Husband education, women's and husband's occupation, economic status (ES), ethnicity, media exposure, family size, urban/rural residence, problematic distance to health facility, region, community literacy, community ES	Having secondary and higher education associated with lower odds of EM (OR: 0.71 [CI: 0.60, 0.85] and 0.25 [0.14, 0.44] respectively; *p* < 0.001)—but not having primary school education (1.02 [0.87, 1.20]), compared to having no formal education	4

Abbreviations: CI, confidence interval; Coeff, coefficient; EM, early marriage; HH, household; HR, hazard ratio; OR, odds ratio; PR, prevalence ratio; RoB, risk of bias; RRR, relative risk ratio; SD, standard deviation; SE, standard error.

### 4.4 H4: Preterm Birth Is Associated With Reduced Cognitive Function

All five systematic reviews found evidence of poorer cognitive function in children born preterm, compared to their term‐born peers (Table 4). With the exception of one study from Belarus, included in the review of Chan et al. (2016) and two studies from India, included in the review of McBryde et al. (McBryde et al. 2020), all the studies analyzed were from high‐income countries.

**TABLE 4:** Summary of systematic reviews on the association of preterm birth with educational outcomes.

Author (year)	Studies	Setting	Exposure outcome(s)	Risk of bias assessment	Main findings
Chan et al. (2016)	*N* = 22	HIC + Belarus	PB: 37–38 weeks (ET), 37–36 weeks (LPT) Cognitive ability: Using validated instruments, for example, Wechsler Intelligence Scale for Children School performance: Verbal/nonverbal IQ, reading/language, and mathematics Educational attainment: Length of completed education Others: Rates of developmental disability and SEN	Data source validity, selection of participants, attrition, reporting bias and measurement of confounders Grading system: Hybrid from Cochrane Handbook Criteria and Newcastle–Ottawa Scale	General cognitive ability: Inverse association between general cognitive ability and gestational age in childhood Limited evidence showed that ET/LPT children had lower verbal/nonverbal IQ compared to term children School performance: LPT and ET had higher chance of having lower school performance, including poorer language. Mixed findings in maths performance LPT and ET predispose to poorer IQ and school performance, lower likelihood of completing secondary and post‐secondary school School attainment: LPT children increased the chance of failing to complete high school while ET adults were less likely to attain post‐secondary school
Moreira et al. (2014)	*N* = 33	HIC	PB was not clearly defined but in the articles found: < 32, 32–36, or < 37 weeks School performance: Structured questionnaire developed by researchers, WRAT‐3, and WJIII	Assessed using Strengthening the reporting of observational studies in epidemiology (STROBE) and Physiotherapy Evidence Database (PEDro) scales	Most studies found that preterm children had some school‐related problem School performance was mostly evaluated by assessing learning domains, for example, maths, reading, and writing
Allotey et al. (2018)	*N* = 74	HIC	PB: very (< 28 weeks), moderate (28–33^+6^ weeks) and late (34–36^+6^ weeks) Cognitive performance, motor skills, academic performance, and behavior differences	Assessed using the Newcastle–Ottawa Quality Assessment Scale	PB children had lower cognitive scores for full scale IQ (SMD: −0.70 [CI: −0.73, −0.66]), performance IQ (SMD: −0.67 [CI: −0.73, −0.60]), and verbal IQ (SMD: −0.53 [CI: −0.60, −0.47]). PB children had lower scores in reading, mathematics and spelling at primary school age, and up to secondary school age, except for mathematics Gestational age at birth accounted for 38%–48% of the observed IQ variance ADHD diagnosed twice as often in PB children (OR: 1.6 [CI: 1.3, 1.8]), with variation by severity of prematurity
Twilhaar et al. (2018)	*N* = 17	HIC	PB: < 37 weeks gestation Academic performance: standardized tests in arithmetic, reading, spelling	Only publication bias assessed (using nonsignificant Egger's test).	Compared to full term children, PB children had lower arithmetic scores (by 0.71 SD; *p* < 0.001), reading scores (0.44 SD; *p* < 0.001) and spelling scores (0.52 SD; *p* < 0.001) and were more likely to receive special educational assistance (RR: 2.85 [CI 2.12, 3.84], *p* < 0.001). Bronchopulmonary dysplasia explained 44% of the variance in academic performance (*p* = 0.006)
McBryde et al. (2020)	*N* = 33	All HIC + India	PB: < 37 weeks gestation Academic performance: standardized tests of reading and mathematics (and associated subskills)	Assessed using the Newcastle–Ottawa Quality Assessment Scale	Compared to children born at term, PB children had lower scores for: reading comprehension (MD: −7.96 [CI: −12.15, −3.76]), applied mathematical problems (−11.41 [−17.57, −5.26]), calculation (−10.57 [−15.62 to −5.52]), decoding (−10.18 [−16.83 to −3.53]), mathematical knowledge (−7.44 [−9.08 to −5.80]), word identification (−7.44 [−9.08 to −5.80]), mathematical fluency (−6.89 [−13.54 to −0.23])

Abbreviations: CI, confidence interval; ET, early term; HIC, high‐income country; IQ, intelligence quotient; LPTI, late preterm infants; LT, late term; MLPT, moderate‐to‐late preterm; OR, odds ratio; *p, p*‐value; PB, preterm birth; RR, risk ratio; SEN, special education needs; SMD, standardized mean difference; WJIII, Woodcock‐Johnson III; WRAT‐3, wide range achievement test 3.

Chan et al. (2016), analyzing 22 studies, found evidence of an inverse association of gestational age with general cognitive ability, with limited evidence that late preterm and early term children had lower verbal and nonverbal IQ scores. Late preterm and early term children demonstrated poorer school performance, and a reduced likelihood of completing secondary and post‐secondary school. Moreira et al. (2014), analyzing 33 studies, found that preterm children had poorer academic performance in 15 of 16 studies. Allotey et al. (2018), performing a meta‐analysis of 74 studies, found that children born preterm had lower IQ scores and lower scores in reading, mathematics and spelling at primary school age, and similar differences up to secondary school age, except for mathematics. Gestational age at birth accounted for 38%–48% of the observed IQ variance. Twilhaar et al. (2018), performing a meta‐analysis of 17 studies, found that preterm children had lower ability in arithmetic, reading and spelling, and were 2.8 times (95% CI 2.1–3.8) more likely to receive special educational assistance. McBryde et al. (2020), performing a meta‐analysis of 33 studies, found that children born preterm had lower scores for reading comprehension, word identification and mathematical abilities.

## 5 Discussion

Studying intergenerational cycles of disadvantage is challenging, as the ideal approach would be a prospective longitudinal cohort that followed the second generation into adulthood and recorded their own reproductive outcomes. Such studies are rare, especially in low‐income settings; hence we adopted an alternative approach, searching the literature systematically for evidence of the three steps that we hypothesized constitute an intergenerational cycle of risk linking girls' early marriage, preterm birth, and school dropout.

Our review found evidence supporting H1 and H3, with the risk of bias generally relatively low. Conversely, we identified only one study that investigated H2, that preterm birth is associated with school dropout in LMICs, and the evidence from this study was inconsistent across the five countries analyzed. However, the systematic reviews, primarily assessing research conducted in HICs, found evidence that preterm birth is associated with poorer cognitive ability and schooling outcomes (H4). Therefore, the concept of the intergenerational cycle appears plausible, but further research is needed.

An increased risk of preterm birth following early marriage (H1) was evident in four studies, two from South Asia where marriage is a near‐universal practice, and two from South America where marriage is less universal. One underlying mechanism may involve early childbearing, as adolescent mothers have an increased risk of delivering a preterm offspring (Fall et al. 2015; Gronvik and Fossgard Sandoy 2018). However, the study from Nepal is particularly informative, as it disentangled the risks of preterm birth associated with early marriage and early reproduction (Miller et al. 2022). The finding that the risk of preterm birth was elevated in primigravidae who had married very early (< 14 years), independent of their age of childbearing, suggests that exposure to psychosocial stress might be part of the underlying mechanism. Further research is needed to understand how both early marriage and early reproduction relate to the risk of preterm birth.

The risk of school dropout in LMICs following preterm birth (H2) was assessed by only one study. This large‐scale analysis, pooling data from five birth cohorts in Brazil, Guatemala, India, the Philippines, and South Africa found that educational attainment was 0.44 years lower (95% CI 0.17–0.71) following preterm birth compared to term birth. However, disaggregating by country, the association was significant only in India and the Philippines. The lack of data available for testing H2 reflects the fact that long‐term prospective longitudinal studies are needed. Only prospective birth cohorts are likely to have assessed both gestational age and school outcomes in adolescence. Research on school‐aged children typically locates children in school, and hence will inherently miss those who have dropped out, even if preterm birth status was collected retrospectively. On a shorter time scale, a systematic review of the predictors of child development below 7 years in LMICs found that compared with term and appropriate for gestational age (AGA) infants, preterm‐AGA infants had 0.14 SD (CI −0.24 to 0.05) and 0.23 SD (CI −0.42 to 0.03) lower cognitive and motor scores, respectively (Sania et al. 2019). However, lacking educational outcomes, this study did not satisfy the inclusion criteria for our review, and the implications of these associations for subsequent school dropout remain unknown.

Systematic reviews of studies from any setting, the vast majority from high‐income settings, supported the hypothesis that preterm birth impairs cognitive capacity and school performance (H4), and increases the likelihood of school dropout. In addition, a large study from Scotland, which was not included in our review as it did not fit the LMIC inclusion criteria, found a near‐linear association of shorter gestation with difficulties in school, whereby each 1‐week reduction in gestational length below 41 weeks was associated with an increased likelihood of the child requiring special educational needs (MacKay et al. 2010). A shorter duration of intrauterine growth disrupts neurodevelopmental processes such as synapse formation and myelination, which may result in neurocognitive deficits (Counsell and Boardman 2005; Vo Van et al. 2022). Moreover, other factors might also contribute; for example children born preterm may remain smaller than their age peers (Christian et al. 2013), and might be held back from starting school (Brown and Pollitt 1996), which could potentially undermine their educational potential (Chen 2015; Jaganath et al. 2015).

Finally, studies from across the Global South (mainly from sub‐Saharan Africa and Asia) provided consistent evidence that school dropout is associated with increased risk of girls' early marriage. The study designs were heterogeneous, making it difficult to assign the direction of causality. Indeed, one study already demonstrated that early marriage and school dropout may represent a “two‐way” street (Marphatia, Wells, et al. 2021). Girls performing poorly in school may be selected by their parents for early marriage, while early marriage may pull girls out of school. Some studies specifically supported the first of these pathways. For example, in Malawi, dropping out of primary school was associated with an increased risk of early marriage (Glynn et al. 2018). Similarly, in India, poor educational performance at 12 years was associated with an increased risk of marriage before 18 years (Kanji et al. 2024), and completing primary school was associated with lower odds of early marriage (Paul 2020). Detailed prospective longitudinal studies are required to increase this evidence base.

While the associations we identified are not determinative at the individual level, and refer rather to increased risks of adverse outcomes following the exposures, the population burden may nevertheless be substantial because each of preterm birth, school dropout and early marriage is common in the Global South. For example, a modeling study projected an assumed effect of preterm birth on educational attainment onto 622 million live births across five birth cohorts, spanning 121 countries (Blakstad et al. 2022). Across all countries combined, the model indicated that reducing preterm birth to a theoretical minimum prevalence of 5.5%, based on evidence from the INTERGROWTH‐21st study, would be associated with a potential gain of 9.8 million school years (95% CI: 1.5–18.4), including 3.66 (0.55, 7.37) million years in South Asia and 3.06 (0.46, 5.92) million in Sub‐Saharan Africa (Blakstad et al. 2022). These results are not expressed in a typical extended period in school at the individual level, but they indicate the potential for a change in one component of the cycle to impact another at scale.

The underlying mechanisms in the hypothesized cycle require further attention. As discussed above, early marriage may increase the risk of preterm birth through pathways such as psychosocial stress, inadequate nutrition, early reproduction or incomplete pelvic growth. The link between preterm birth and school dropout might involve direct detrimental effects on brain growth or function (MacKay et al. 2010), or it could reflect a common underlying driver. For example, a study by Huang et al. (2021) assessed heavy metal exposures in the cord blood of Bangladeshi babies and their associations with preterm birth. Titanium, arsenic, and barium exposure all predicted preterm birth, with an increased element risk score almost tripling the odds of preterm birth (OR = 2.72, 95% CI: 1.57–4.69). These metals may also impair brain development (C. Liang et al. 2020). Interestingly, Huang et al. found a significant moderation effect of child marriage on their element risk score; an association between cord blood element load and preterm birth was only found in women who married before 18 years. A study by Rahman et al. (2018) also conducted in Bangladesh found that both early marriage and arsenic exposure were associated with preterm birth, and that a lowering of pregnancy weight gain mediated these associations. Therefore, early marriage may increase susceptibility to other biological stressors.

Finally, although school dropout may precipitate early marriage (as families may elect to marry a daughter early following poor school performance), the reverse scenario may also occur, as girls may be prevented from attending school following their early marriage. A study from India found evidence for both pathways in the same community, and also that a small minority of early‐married girls were still attending school (Marphatia, Wells, et al. 2021). Moreover, schooling in some societies is directly related to marriage decisions, as greater education increases the value of an incoming bride to the marital household, and also affects the amount of dowry (Jeffery and Jeffery 1994). Therefore, the association of educational attainment and marriage is complex and merits further attention.

Importantly, common factors precipitating both school dropout and early marriage may also lie outside the family and household domain. For example, in the Democratic Republic of Congo, civil conflict drove girls to drop out of school, which was in turn associated with earlier sexual debut and adolescent marriage, and ultimately adolescent motherhood (Mugisho 2024).

Our findings are especially relevant to settings such as South Asia where marriage is near‐universal, as efforts to delay marriage have the greatest potential to disrupt this intergenerational cycle. In South America, where marriage is less obligatory, unmarried women have an increased risk of having a child born preterm compared to married women (Urquia, Batista, Cunha Cardoso, et al. 2022; Urquia, Batista, Grandi, et al. 2022), whereas among married women, earlier marriage was associated with increased risk.

Our findings are consistent with the hypothesis that early marriage depletes maternal capital, an umbrella term for components of maternal phenotype that promote the capacity for investment in offspring (J. C. Wells 2010). While the evidence that we have reviewed supports the intergenerational cycle that we hypothesized, in reality the components we focused on are part of a broader intergenerational cycle that also includes the detrimental effects of malnutrition and poverty on maternal capital. In Brazil and India, we have shown that depletions in both biological and social components of maternal capital impair outcomes of the offspring, and moreover increase the likelihood of the same depletions in maternal capital recurring in the next generation, when the offspring reach adulthood and start reproducing (Marphatia, Wells, Reid, Bhalerao, and Yajnik 2025; J. C. Wells et al. 2019). Complementary to the pathways we have explored here, early marriage has also been associated with infant undernutrition (Raj et al. 2010; J. C. Wells et al. 2022), which in turn has been associated with school dropout (Katoch et al. 2022).

Our review adds to growing awareness of the intricate links between behaviors that might appear strongly cultural (household decisions about schooling and marriage), and biological traits (brain development) that are sensitive to diverse physical factors such as malnutrition, pollution, and the stress response. We need to move beyond disciplinary silos, whereby education is seen only as a school‐based issue, and marriage as only a household transactional issue, to understand that the variability in such decisions has much deeper roots that are embedded in biological mechanisms.

Our study had some strengths, including the use of systematic searches to obtain all relevant evidence, and the use of specific search terms that were able to identify a number of studies in both biomedical and social science literature. The risk of bias in the studies identified was generally low. However, our approach also had several limitations. All empirical studies were observational, and hence cannot directly demonstrate causation. Each of [Link], [Link], [Link] demonstrated heterogeneity in how the exposure and outcome were categorized, preventing any meta‐analysis. The number of available publications was low for H1 and especially for H2. For H2 and H4, the evidence relates to all preterm births (involving multiple risk pathways), and does not relate specifically to preterm births associated with early marriage. For H3, only a minority of studies reliably indicated the direction of association between lower educational attainment and early marriage. There is a need for more prospective research on [Link], [Link], [Link], in particular on the long‐term consequences of preterm birth in LMICs. Research on preterm birth and cognitive function may not differentiate by child sex, and research on brain development in LMICs has shown that males tend to have lower scores than females (McCoy et al. 2016). Heterogeneity in reference and comparison groups meant we were unable to perform any meta‐analysis. For the two hypotheses that relate to preterm birth, the majority of evidence relates to later preterm deliveries, hence our review reflects this. However, this scenario is consistent with the global distribution of the exposure. The burden of late preterm infants is significantly higher in LMICs compared to HICs (March of Dimes, PMNCH, Save the Children, and WHO 2012), and late preterm infants have higher survival rates compared to very or extremely preterm infants in LMICs, due to limited availability of medical care (> 50% vs. 10%) (Blencowe et al. 2013), hence most children born preterm are also late preterm. We note that very or moderate preterm children appear to have distinct educational outcomes compared to late preterm children (Loftin et al. 2010; Smyrni et al. 2021), hence the associations we have described for late preterm births might be different in these other groups.

## 6 Conclusion

We found some evidence from LMICs for each of the three steps in a biosocial intergenerational cycle of risk—that girls' early marriage may increase the risk of preterm birth, that preterm birth may be associated with reduced educational attainment, in part through effects on cognitive function, and that school dropout may increase the risk of girls' early marriage. However, the evidence base is currently limited, particularly for H2, and only a minority of studies analyzed for H3 indicate the hypothesized direction of association, namely that school dropout increases the risk of early marriage. While the risk of bias in empirical studies was generally low, there is a need for more prospective longitudinal studies to test these hypotheses more rigorously, and quantify the magnitude of the associations.

Acknowledging the limitations of the evidence base at this stage, we propose that these relationships may contribute to a broader intergenerational cycle, involving a larger number of traits and mechanisms such as poverty and stunting. Breaking such intergenerational cycles will require political will, as the full benefits will inevitably take time to emerge. For this particular pathway, as preterm birth is difficult to prevent at the individual level, the more promising opportunities lie in promoting girls' education across the whole range of educational ability, and preventing early marriage.

## Funding

The authors have nothing to report.

## Ethics Statement

The authors have nothing to report.

## Conflicts of Interest

The authors declare no conflicts of interest.

## Supporting information


**Data S1:** Supporting Information.

## Data Availability

Data sharing not applicable to this article as no datasets were generated or analysed during the current study.
