# Supplementary material for: Early Marriage, Preterm Birth, and School Dropout: An Intergenerational Cycle of Risk?
Source: Am J Hum Biol. 2025 Dec 15;37(12):e70177. doi: 10.1002/ajhb.70177 (PMC12703574; doi:10.1002/ajhb.70177)
Supplement: Supplementary file 1 — Data S1: Supporting Information. [file AJHB-37-e70177-s001.pdf]

Early marriage, preterm birth and school dropout: an intergenerational cycle of risk? Supplementary online material

Risk of bias assessment for H1

Reference:	Miller et al. 2021	Pandya & Bhanderi 2015	Urquia et al. 2022a	Urquia et al. 2022b
Study design:	Longitudinal	Cohort	Cohort	Cohort
**CRITERIA FOR CROSS-SECTIONAL STUDIES (MODIFIED OTTAWA SCALE - 9 POINTS MAX)**				
**Selection (maximum 2 points)**				
1) Is the sample truly representative of the target population (i.e. were all subjects used or random-sampling and appropriate weighting used (yes or somewhat = 1 point)		0	1	1
2) Is the sample size justified and satisfactory (yes = 1 point)		0	1	1
**Assessment of exposure and outcome (maximum 4 points)**				
3) Were exposures assessed using a gold-standard or validated assessment tool (yes = 2 points / no but the tool is widely used and/or described = 1 point)		0	1	1
4) Were outcomes assessed using a gold-standard or validated assessment tool (yes = 2 points / no but the tool is widely used and/or described = 1 point)		0	2	2
**Confounding factors (maximum 3 points)**				
1) The study estimates are adjusted for all the most important confounders, and not adjusted for potential mediators (2 points) OR most important confounders and potential mediators (1 point) OR some confounders but not all the most important (1 point) OR none of the important confounders OR no description provided		0	2	2
2) Most of the confounding factors assessed using gold standard or validated or acceptable tools (1 point) OR not OR no confounding controlled for.		0	1	1
TOTAL		**0**	**8**	**8**
**CRITERIA FOR CASE CONTROL STUDIES (ORIGINAL OTTAWA SCALE- 9 POINTS MAX)**				
**Selection (maximum 4 points)**				
1) Is the case definition adequate? Yes with independent validation (1 point) OR yes record linkage or self-report OR no description				
2) Representativeness of the cases: are they consecutive or obviously representative of cases (1 point) or is there potential for selection biases or is it not stated				
3) Selection of Controls: are controls from the community (1 point) or hospital setting or not described				
4) Definition of Controls: do the controls have no history of disease (1 point) or is there no description of source				
**Comparability (maximum 2 points)**				
Are cases and controls comparable on the basis of the design or analysis? The study controls for the most important factor (1 point) and/or additional factors (1 point) or nothing/not described.				
**Exposure (maximum 3 points)**				
1) Ascertainment of exposure: is it a secure record (e.g. surgical) and/or a structured interview where blind to case/control status (1 point) or not blinded interview or self-report or medical record only or not described				
2) Same method of ascertainment for cases and controls: yes (1 point) or no				
3) Non response rate: same rate for both groups (1 point) or non-respondents described or rate differs and no designation				
TOTAL				
**CRITERIA FOR COHORT STUDIES (ORIGINAL OTTAWA SCALE - 9 POINTS MAX)**				
**Selection (maximum 4 points):**				
1) Is the exposed cohort truly representative of the average exposure in the community (i.e. were all subjects used or random-sampling and appropriate weighting used (yes or somewhat = 1 point) OR selected group OR no description	1			
2) Is the non-exposed cohort drawn from the same community as the exposed cohort (1 point) OR a different source OR not described	1			
3) Was the exposure assessed using secure records or structured interview (1 point) OR written self-report OR not described	1			
4) Was it demonstrated that the outcome of interest was not present at start of study? Yes (1 point) OR no	1			
**Comparability (maximum 2 points)**				
Are subjects in different outcome categories comparable, either based on study design or statistical analysis (are confounding factors controlled for) (the study controls for the most important confounders = 2 points, the study controls partially for confounding factors = 1 point)	2			
**Outcome (maximum 3 points)**				
1) Were outcomes assessed adequately, e.g. through independent blind assessment or record linkage (1 point) OR self-report OR not described	0			
2) Was follow-up long enough for outcomes to occur? Yes (1 point) OR no	1			
3) Was follow-up of cohorts adequate? Yes (complete follow-up, all subjects accounted for OR low loss to follow-up, unlikely to introduce bias or correctly described - 1 point) OR no (high loss and/or no description of those lost)	0			
TOTAL	**7**			

Risk of bias assessment for H2

**Reference:**	**Stein et al. 2013**
**Study design:**	Cohort
**CRITERIA FOR CROSS-SECTIONAL STUDIES (MODIFIED OTTAWA SCALE - 9 POINTS MAX)**	
**Selection (maximum 2 points):**	
1) Is the sample truly representative of the target population (i.e. were all subjects used or random-sampling and appropriate weighting used (yes or somewhat = 1 point)	
2) Is the sample size justified and satisfactory (yes = 1 point)	
**Assessment of exposure and outcome (maximum 4 points)**	
3) Were exposures assessed using a gold-standard or validated assessment tool (yes = 2 points / no but the tool is widely used and/or described = 1 point)	
4) Were outcomes assessed using a gold-standard or validated assessment tool (yes = 2 points / no but the tool is widely used and/or described = 1 point)	
**Confounding factors (maximum 3 points)**	
1) The study estimates are adjusted for all the most important confounders, and not adjusted for potential mediators (2 points) OR most important confounders and potential mediators (1 point) OR some confounders but not all the most important (1 point) OR none of the important confounders OR no description provided	
2) Most of the confounding factors assessed using gold standard or validated or acceptable tools (1 point) OR not OR no confounding controlled for.	
TOTAL	
**CRITERIA FOR CASE CONTROL STUDIES (ORIGINAL OTTAWA SCALE- 9 POINTS MAX)**	
**Selection (maximum 4 points)**	
1) Is the case definition adequate? Yes with independent validation (1 point) OR yes record linkage or self-report OR no description	
2) Representativeness of the cases: are they consecutive or obviously representative of cases (1 point) or is there potential for selection biases or is it not stated	
3) Selection of Controls: are controls from the community (1 point) or hospital setting or not described	
4) Definition of Controls: do the controls have no history of disease (1 point) or is there no description of source	
**Comparability (maximum 2 points)**	
Are cases and controls comparable on the basis of the design or analysis? The study controls for the most important factor (1 point) and/or additional factors (1 point) or nothing/not described.	
**Exposure (maximum 3 points)**	
1) Ascertainment of exposure: is it a secure record (e.g. surgical) and/or a structured interview where blind to case/control status (1 point) or not blinded interview or self-report or medical record only or not described	
2) Same method of ascertainment for cases and controls: yes (1 point) or no	
3) Non response rate: same rate for both groups (1 point) or non-respondents described or rate differs and no designation	
TOTAL	
**CRITERIA FOR COHORT STUDIES (ORIGINAL OTTAWA SCALE - 9 POINTS MAX)**	
**Selection (maximum 4 points):**	
1) Is the exposed cohort truly representative of the average exposure in the community (i.e. were all subjects used or random-sampling and appropriate weighting used (yes or somewhat = 1 point) OR selected group OR no description	1
2) Is the non-exposed cohort drawn from the same community as the exposed cohort (1 point) OR a different source OR not described	1
3) Was the exposure assessed using secure records or structured interview (1 point) OR written self-report OR not described	1
4) Was it demonstrated that the outcome of interest was not present at start of study? Yes (1 point) OR no	1
**Comparability (maximum 2 points)**	
Are subjects in different outcome categories comparable, either based on study design or statistical analysis (are confounding factors controlled for) (the study controls for the most important confounders = 2 points, the study controls partially for confounding factors = 1 point)	1
**Outcome (maximum 3 points)**	
1) Were outcomes assessed adequately, e.g. through independent blind assessment or record linkage (1 point) OR self-report OR not described	0
2) Was follow-up long enough for outcomes to occur? Yes (1 point) OR no	1
3) Was follow-up of cohorts adequate? Yes (complete follow-up, all subjects accounted for OR low loss to follow-up, unlikely to introduce bias or correctly described - 1 point) OR no (high loss and/or no description of those lost)	1
TOTAL	**7**

Risk of bias assessment for H3

Reference:	Bengesai et al. 2021	Bhan et al. 2019	Cameron et al. 2023	Fang et al. 2024	Glick et al. 2015	Glynn et al. 2018	Kanji et al. 2024	Kumar et al. 2023	Lami et al. 2024	Liang & Yu 2022	Marphatia et al. 2021	Marphatia et al. 2020	Paul 2019	Paul 2020	Prakash et al. 2017	Roy & Chouhan 2021	Sagalova et al. 2021	Sekine & Hodgkin 2017	Singh et al. 2024	Zegeye et al. 2021
**Study design:**	Cohort (Co)	Co	Co	Co	Co	Co	Co	Co	CS	Panel	Co	Longitudinal	CS	CS	CS	CS	CS	CS	CS	CS
**CRITERIA FOR CROSS-SECTIONAL STUDIES (MODIFIED OTTAWA SCALE - 9 POINTS MAX)**																				
**Selection (maximum 2 points):**																				
1) Is the sample truly representative of the target population (i.e. were all subjects used or random-sampling and appropriate weighting used (yes or somewhat = 1 point)	1			1	0				1				1	1	1	1	1	1	1	1
2) Is the sample size justified and satisfactory (yes = 1 point)	1			1	1				1				1	1	1	1	1	1	1	1
**Assessment of exposure and outcome (maximum 4 points)**																				
3) Were exposures assessed using a gold-standard or validated assessment tool (yes = 2 points / no but the tool is widely used and/or described = 1 point)	1			1	1				1				1	1	1	1	1	1	1	1
4) Were outcomes assessed using a gold-standard or validated assessment tool (yes = 2 points / no but the tool is widely used and/or described = 1 point)	1			1	1				0				1	1	1	1	1	1	1	1
**Confounding factors (maximum 3 points)**																				
1) The study estimates are adjusted for all the most important confounders, and not adjusted for potential mediators (2 points) OR most important confounders and potential mediators (1 point) OR some confounders but not all the most important (1 point) OR none of the important confounders OR no description provided	2			1	2				0				1	0	1	0	1	1	0	0
2) Most of the confounding factors assessed using gold standard or validated or acceptable tools (1 point) OR not OR no	1			1	1				0				1	0	1	0	0	1	0	0

confounding controlled for.																				
TOTAL	7			**7**	**6**				**3**				**6**	**4**	**6**	**4**	**5**	**6**	**4**	**4**
**CRITERIA FOR CASE CONTROL STUDIES (ORIGINAL OTTAWA SCALE- 9 POINTS MAX)**																				
**Selection (maximum 4 points)**																				
1) Is the case definition adequate? Yes with independent validation (1 point) OR yes record linkage or self-report OR no description																				
2) Representativeness of the cases: are they consecutive or obviously representative of cases (1 point) or is there potential for selection biases or is it not stated																				
3) Selection of Controls: are controls from the community (1 point) or hospital setting or not described																				
4) Definition of Controls: do the controls have no history of disease (1 point) or is there no description of source																				
**Comparability (maximum 2 points)**																				
Are cases and controls comparable on the basis of the design or analysis? The study controls for the most important factor (1 point) and/or additional factors (1 point) or nothing/not described.																				
**Exposure (maximum 3 points)**																				
1) Ascertainment of exposure: is it a secure record (e.g. surgical) and/or a structured interview where blind to case/control status (1 point) or not blinded interview or self-report or medical record only or not described																				
2) Same method of ascertainment for cases and controls: yes (1 point) or no																				
3) Non response rate: same rate for both groups (1 point) or non respondents described or rate differs and no designation																				

TOTAL																				
**CRITERIA FOR COHORT STUDIES (ORIGINAL OTTAWA SCALE - 9 POINTS MAX)**																				
**Selection (maximum 4 points):**																				
1) Is the exposed cohort truly representative of the average exposure in the community (i.e. were all subjects used or random-sampling and appropriate weighting used (yes or somewhat = 1 point) OR selected group OR no description		1	1			0	1	1		0	0	0								
2) Is the non exposed cohort drawn from the same community as the exposed cohort (1 point) OR a different source OR not described		1	1			1	1	1		1	1	1								
3) Was the exposure assessed using secure records or structured interview (1 point) OR written self-report OR not described		1	1			1	1	1		1	1	1								
4) Was it demonstrated that the outcome of interest was not present at start of study? Yes (1 point) OR no		1	1			1	1	1		1	1	1								
**Comparability (maximum 2 points)**																				
Are subjects in different outcome categories comparable, either based on study design or statistical analysis (are confounding factors controlled for) (the study controls for the most important confounders = 2 points, the study controls partially for confounding factors = 1 point)		2	2			2	2	0		2	1	2								
**Outcome (maximum 3 points)**																				
1) Were outcomes assessed adequately, e.g. through independent blind assessment or record linkage (1 point) OR self-report OR not described		0	0			0	0	0		1	0	0								

2) Was follow-up long enough for outcomes to occur? Yes (1 point) OR no		1	1			1	1	1		1	1	1								
3) Was follow-up of cohorts adequate? Yes (complete follow-up, all subjects accounted for OR low loss to follow-up, unlikely to introduce bias or correctly described - 1 point) OR no (high loss and/or no description of those lost)		0	0			0	0	1		0	1	1								
TOTAL		**7**	**7**			**6**	**7**	**6**		**7**	**7**	**7**								
